# A Relationship: Word Alignment, Phrase Table, and Translation Quality

**DOI:** 10.1155/2014/438106

**Published:** 2014-04-16

**Authors:** Liang Tian, Derek F. Wong, Lidia S. Chao, Francisco Oliveira

**Affiliations:** Natural Language Processing & Portuguese-Chinese Machine Translation Laboratory, Department of Computer and Information Science, University of Macau, Macau

## Abstract

In the last years, researchers conducted several studies to evaluate the machine translation quality based on the relationship between word alignments and phrase table. However, existing methods usually employ ad-hoc heuristics without theoretical support. So far, there is no discussion from the aspect of providing a formula to describe the relationship among word alignments, phrase table, and machine translation performance. In this paper, on one hand, we focus on formulating such a relationship for estimating the size of extracted phrase pairs given one or more word alignment points. On the other hand, a corpus-motivated pruning technique is proposed to prune the default large phrase table. Experiment proves that the deduced formula is feasible, which not only can be used to predict the size of the phrase table, but also can be a valuable reference for investigating the relationship between the translation performance and phrase tables based on different links of word alignment. The corpus-motivated pruning results show that nearly 98% of phrases can be reduced without any significant loss in translation quality.

## 1. Introduction

One of the best performing translation systems in Statistical Machine Translation (SMT) nowadays is the phrase-based model, which takes continual word sequences as translation units [1, 2].

The fundamental data structure in phrase-based models is a table of phrase pairs with associated scores which may come from probability distributions. Until now, several methods to extract phrase pairs from a parallel corpus have been proposed, such as using a probabilistic model [3], pattern mining methods [4], matrix factorization [5], heuristic-based method [6–9], MBR-based method [10], and model-based method [11]. However, most commonly, this table is acquired from word alignments [12–15], which exhaustively enumerates all phrases up to a certain length consistent with the alignment [16].

When talking about the relationship between machine translation and word alignment or phrase table, researchers seek for better translation performance from at least two independent research efforts. On the one hand, different processes of interfering word alignments were studied for better translation results. Some researchers have shown that translation quality depends on word alignment quality [7, 17]. Another group of researchers hold an opposite point of view: significant decreases AER (alignment error rate) will not always result in significant increases the translation quality [8, 18, 19]. Recently, researchers in [20] made a systematic study and pointed out that alignments can improve the translation performance depending on the SMT systems and the type of corpus used.

On the other hand, phrase table pruning techniques were applied for better machine performance without losing the overall quality. Threshold values are considered to reduce the phrase table size, which are usually related to* absolute* scores of phrase pairs in the phrase table or* relative* scores between the phrase tables sharing their source phrases. Authors in [21, 22] proposed a significant testing in order to select only those phrase pairs which are the more-than-random occurring ones in the training corpus. References [23, 24] exploited the same idea for the hierarchical SMT. References [25, 26] considered usage statistics of phrase pairs from translating sample data, which are based on how often a phrase pair was considered during decoding and how often it was used in the best translation. In [27, 28], the researchers adapted* triangulation* approach to prune the phrase table, which requires additional bilingual corpora. Authors in [10, 29, 30] modified the phrase extraction methods in order to reduce the phrase table size. In [31, 32], they introduced an* entropy*-*based* pruning criterion to prune the generated phrase tables.

Both studies tried to reduce the spurious results (errors or redundancies) to investigate the affection to the final translation performance based on different empirical statistics. In other words, many existing methods usually employ ad hoc heuristics without theoretical proof. We try to make a study on the relationship among word alignments, phrase table, and translation quality originally from a mathematical perspective proposed in this paper. The equation indicates that it is necessary to improve the quality of word alignment, even if the alignment does not explicitly connect to the translation quality. In particular, by lowering the quality of alignment, the affection to the translation is very minor. In this paper, two extreme cases are that the* best* and the* worst* quality of word alignments are used to illustrate the situation. Experiment results show that the quality of word alignment will affect the translation performance a lot measured by BLEU [33].

The contributions of this paper mainly focus on two aspects: (1) formulating the relationships among word alignment, phrase table, and machine translation; the equations indicate that the better quality of the word alignment, the fewer the phrases will be, and the better translation performance both on phrase-level and finer granularity levels; (2) corpus-motivated pruning techniques to prune the default large phrase table, which can remove nearly 85% of all the phrase pairs without hurting the performance of the translation.

The rest of the paper is organized as follows.  Section 2 reviews the related works and background that will be used in this paper.  Section 3 describes the relationship details among the word alignment, phrase table, and machine translation from a mathematical perspective. And a corpus-motivated pruning method is introduced here.  Section 4 describes the conducted experiments and presents the obtained results. The paper concludes with a summary and discussions of the results.

## 2. Related Works and Background

Our work is based on the dominant method to obtain a phrase table from word alignment, which trained from the EM (Expectation Maximization) algorithm. To extract the phrases from the word alignment, EM algorithm will be utilized to train the bilingual corpus for several iterations, and then phrase pairs that are* consistent* with this word alignment will be extracted. Most of the current Phrase-Based SMT systems rely on the GIZA++ implementation of the IBM Models [34] to produce word alignments, running the algorithm in both directions, source to target and target to source. Various heuristics can then be applied to obtain a* symmetrized* alignment from those two. Most of them, such as* grow-diag-final-and*  [8] start from the* intersection* of the two word alignments and enrich it with alignment points from the* union*.

Suppose a phrase pair is represented by $\left(\tilde{f},\tilde{e}\right)$ consistent with an alignment *a*, if all words *f*_1_,…, *f*_*j*_,…, *f*_*J*_ in $\tilde{f}$ that have alignment points in *a* have these with words *e*_1_,…, *e*_*i*_,…, *e*_*I*_ in $\tilde{e}$ and vice versa. More formal definition of the consistent with the word alignment can be described as follows [35].

$\left(\tilde{f},\tilde{e}\right)$ consistent with *a*⇔

(1) $$\begin{array}{c} \forall e_{i}\in \tilde{e}:\left(e_{i},f_{i}\right)\in a⟹f_{i}\in \tilde{f},\\ \forall f_{i}\in \tilde{f}:\left(e_{i},f_{i}\right)\in A⟹e_{i}\in \tilde{e},\\ \exists e_{i}\in \tilde{e},\;f_{i}\in \tilde{f}:\left(e_{i},f_{i}\right)\in a. \end{array}$$

This algorithm indicates that all the phrases will be extracted if the biphrases $\left(\tilde{f},\tilde{e}\right)$ alone with their alignment *a*′ satisfy the following two conditions [36]:

- $\tilde{e}$ and $\tilde{f}$ are consecutive word subsequences in the target sentence *e* and source sentence *f*, respectively, and neither of them is longer than *k* words;
- *a*′, the alignment between the words of $\tilde{f}$ and $\tilde{e}$ induced by *a*, contains at least one link.

For the relationship between phrase table and machine translation, most researchers talk about the effects based on different sizes of phrase table, which concerns the phrase table pruning techniques. There are mainly three pruning methods:* statistical-based*,* significance testing,* and* entropy-based* methods. For more details, there are* absolute* and* relative* pruning methods in the* statistical-based* method. The* absolute* pruning methods rely only on the statistics of a single phrase pair $\left(\tilde{f},\tilde{e}\right)$. They may prune all translations of a source phrase that fall below a threshold according to the count $N\left(\tilde{f},\tilde{e}\right)$ or the probability $p\left(\tilde{e}|\tilde{f}\right)$ of the phrase pair $\left(\tilde{f},\tilde{e}\right)$ as shown in (2). Hence, they are independent of other phrases in the phrase table

(2) $$\begin{array}{c} N\left(\tilde{f},\tilde{e}\right)<\tau_{c},\\ p\left(\tilde{e}\;|\;\tilde{f}\right)<\tau_{p}. \end{array}$$

However, the* relative* pruning methods consider the full set of target phrases for a specific source phrase $\tilde{f}$. Given a pruning threshold *τ*_*t*_, a phrase pair $\left(\tilde{f},\tilde{e}\right)$ is discarded if

(3) $$\begin{array}{c} p\left(\tilde{e}\;|\;\tilde{f}\right)<\tau_{t}\;\underset{\tilde{e}}{\max}\left\{p\left(\tilde{e}\;|\;\tilde{f}\right)\right\}. \end{array}$$

Both the* significance testing* and* entropy-based* method are trying to find a threshold *τ* to reduce the size of the generated phrase table.

For the* entropy-based* approach, in [32], they used conditional* Kullback*-*Leibler* divergence [37] to measure the pruned model $p^{\prime }\left(\tilde{e}|\tilde{f}\right)$, which is to be as similar as possible to the original model $p\left(\tilde{e}|\tilde{f}\right)$. Then a pruning threshold *τ*_*E*_ can be chosen and phrase pairs with a contribution to the relative entropy below that threshold are pruned if

(4) $$\begin{array}{c} p\left(\tilde{e}\;|\;\tilde{f}\right)\left[\log p\left(\tilde{e}\;|\;\tilde{f}\right)-\log p^{\prime }\left(\tilde{e}\;|\;\tilde{f}\right)\right]<\tau_{E}. \end{array}$$

For the* significance testing* pruning approach, it heavily depends on a *P*-value. The value is calculated from two by two* contingency* tables, $CT\left(\tilde{f},\tilde{e}\right)$, as shown in Table 1, which can be used to represent the relationship between $\tilde{f}$ and $\tilde{e}$, where the $N(\tilde{f},\tilde{e})$ is defined as the number of parallel sentences that contain one or more occurrences of $\tilde{f}$ on the source side and $\tilde{e}$ on the target side; $N(\tilde{f})$ is the number of parallel sentences that contain one or more occurrences of $\tilde{f}$ on the source side. $N(\tilde{e})$ is the number of parallel sentences that contain one or more occurrences of $\tilde{e}$ on the target side; and *N* is the number of parallel sentences.

Following* Fisher's exact test*, the probability of the contingency table via the hyper geometric distribution can be calculated:

(5) $$\begin{array}{c} p_{h}\left(k\right)=\frac{\left(\begin{array}{c} N\left(\tilde{f}\right)\\ N\left(\tilde{f},\tilde{e}\right) \end{array}\right)\left(\begin{array}{c} N-N\left(\tilde{f}\right)\\ N\left(\tilde{e}\right)-N\left(\tilde{f},\tilde{e}\right) \end{array}\right)}{\left(\begin{array}{c} N\\ N\left(\tilde{e}\right) \end{array}\right)}. \end{array}$$

Then the *P*-value can be then calculated by summing probabilities for tables that have a larger $N(\tilde{f},\tilde{e})$:

(6) $$\begin{array}{c} P\text{-value}\left(CT\left(\tilde{f},\tilde{e}\right)\right)=\overset{\min(N(\tilde{f}),N(\tilde{e}))}{\underset{k=N(\tilde{f},\tilde{e})}{\sum }}p_{h}\left(k\right). \end{array}$$

A phrase pair will be discarded if the *P*-value is greater than *τ*_*F*_ as shown in (7)

(7) $$\begin{array}{c} P\text{-value}\left(CT\left(\tilde{f},\tilde{e}\right)\right)=\overset{\min\left(N(\tilde{f}),N(\tilde{e})\right)}{\underset{k=N(\tilde{f},\tilde{e})}{\sum }}p_{h}\left(k\right)>\tau_{F}. \end{array}$$

Generally speaking, the goal of the* entropy-based* method is to remove the* redundant* phrases, whereas the other approaches are to try to remove the* low-quality* or* unreliable* phrases.

The knowledge introduced above will be used during our discussions on the relationships among word alignment, phase table, and machine translation. More details will be shown in Section 3.

## 3. Word Alignment & Phrase Table & Machine Translation

In this section, we can see from a theoretical aspect that the quality of the underlying word alignments has a strong influence both on performances of phrases table and phrase-based SMT system.

### 3.1. Word Alignment & Phrase Table

As introduced in the previous section, there are some direct relationships between phrase table and word alignments. In this part, the relationship between the word alignments and extracted phrases is formulated to estimate the size of the target phrase table. Two different word alignment cases are considered for the relations. Firstly, the phrases are extracted from the* full word alignment* links of parallel sentences, which are the best word alignments. Secondly, phrases based on a* single link* of word alignment will be made an investigation, which can be treated as the worst alignment information.

#### 3.1.1. Phrases Extraction from Full Word Alignment

Suppose a source sentence *f*_1_^*J*^ = *f*_1_ *f*_2_ ⋯ *f*_*J*_ will be translated into a target sentence *e*_1_^*I*^ = *e*_1_ *e*_2_ ⋯ *e*_*I*_, and only one target word is allowed to align to one or multiple source words. Where *I* and *J* are the length of the target and source sentences, respectively; *i* and *j* are the positions of words in the target and source sentences.

If each target word *e*_*i*_ has at least one alignment link against the word in source text, named as* full word alignment*, fewest phrase pairs can be extracted.

**Figure 1:**
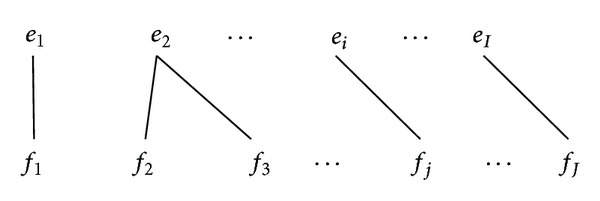
Full word alignments.

For the first case, considering the alignment points in Figure 1, each word *e*_*i*_ in the target sentence has at least one alignment point, and there is no* crossing word alignment*, which means words are monotonically aligned. Starting with the first word *e*_1_ in the target sentence, the phrase pairs with possible extent can be extracted, for example, (*e*_1_ | *f*_1_),   (*e*_1_ *e*_2_ | *f*_1_ *f*_2_ *f*_3_),   (*e*_1_ *e*_2_ ⋯ *e*_*i*_ | *f*_1_ *f*_2_ *f*_3_ ⋯ *f*_*j*_), (*e*_1_ *e*_2_ ⋯ *e*_*i*_ ⋯ *e*_*I*_ | *f*_1_ *f*_2_ *f*_3_ ⋯ *f*_*j*_ ⋯ *f*_*J*_), and so forth, where the total number of *I* phrase pairs can be obtained. Starting with the second word *e*_2_ in the target sentence, *I* − 1 phrase pairs can be extracted, for example, (*e*_2_ | *f*_2_ *f*_3_), (*e*_2_ ⋯ *e*_*i*_ | *f*_2_ *f*_3_ ⋯ *f*_*j*_),   (*e*_2_ ⋯ *e*_*i*_ ⋯ *e*_*I*_ | *f*_2_ *f*_3_ ⋯ *f*_*j*_ ⋯ *f*_*J*_), and so on. Thus, the total number of phrase pairs that extracted from the fully aligned sentences can be defined as

(8) $$\begin{array}{c} I+\left(I-1\right)+\cdots +1=\frac{I\left(I+1\right)}{2}. \end{array}$$

**Figure 2:**
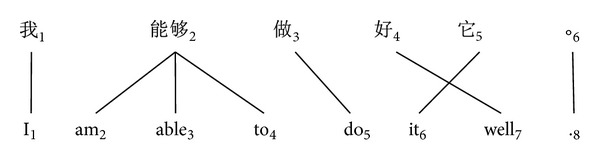
An example of nonmonotonic alignments where alignment links are crossing between parallel sentences.

Actually, the previous alignment type cannot deal with the word alignment that contains the* cross-alignment*, as shown in Figure 2, where the fourth and the fifth Chinese words are cross-aligned with words in source sentence. According to (8), there should be 21 possible phrase pairs. However, only 18 phrases are generated by the conventional phrase extraction algorithm.

**Figure 3:**
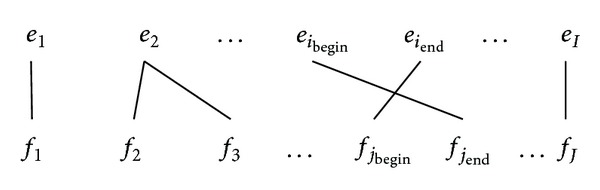
Alignments with a cross link of neighboring words.

The general form of above alignments is illustrated in Figure 3, where the cross-alignments take place between the neighboring words *e*_*i*_begin__ and *e*_*i*_end__; that is, *i*_end_ = *i*_begin_ + 1. Any phrase pairs that fall between the bounds of cross-aligned words cannot be extracted in line with (8). That is, (*i*_begin_ − 1) + (*I* − *i*_end_) phrases should be excluded:

(9) $$\begin{array}{c} N_{\text{ca}}=\left(i_{\text{begin}}-1\right)+\left(I-i_{\text{end}}\right). \end{array}$$

So if we define the reduced phrases as *N*_ca_ (number of cross-alignments), a more practical equation can be revised as

(10) $$\begin{array}{c} N_{\min}=\frac{I\left(I+1\right)}{2}-N_{\text{ca}}. \end{array}$$

If there is more than one cross-alignment as illustrated in Figure 3, the total number of extracted phrases can be generalized as

(11) $$\begin{array}{c} N_{\min}=\frac{I\left(I+1\right)}{2}-\overset{m}{\underset{k=1}{\sum }}\left[\left(i_{\text{begin}}^{k}-1\right)+\left(I-i_{\text{end}}^{k}\right)\right]. \end{array}$$

Reduced phrase pairs are determined by the cross-alignment point, *i*_begin_ and *i*_end_. *i*_begin_^*k*^ represents the start position and *i*_end_^*k*^ is the last position of the *k*th pair words (bounded by the crossing links), and *m* is the total number of cross-alignments.

**Figure 4:**
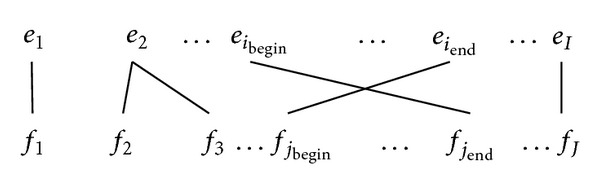
Alignments with a cross link involving distant words.

Figure 3 illustrates the situation that there is not any word between the crossing dependencies. However, in reality, there will be any words in between the cross words, as illustrated in Figure 4.

For the third case, words between *e*_*i*_begin__ and *e*_*i*_end__ may have similar alignment situation as that illustrated in (9). Thus, the following equation can be derived to calculate, *N*_*r*_, the exact number of phrase pairs that cannot be generated. Consider

(12) $$\begin{array}{c} N_{r}=\overset{m}{\underset{k=1}{\sum }}\overset{i_{\text{end}}^{k}-i_{\text{end}}^{k}-1}{\underset{\alpha =0,\beta =0}{\sum }}\left[\left(i_{\text{begin}}^{k}+\alpha -1\right)+\left(I-i_{\text{end}}^{k}+\beta \right)\right], \end{array}$$

where *α* indicates the shift word position after the first cross word *i*_begin_^*k*^ and *β* indicates the word position before the end cross word *i*_end_^*k*^. And *α*,  *β* will not be greater than the number of words between the first and the end cross word.

Finally, the number of phrase pairs that can be extracted from the* full word alignments* measured in the* target* side can be described by

(13) $$\begin{array}{c} N_{\text{min-}t}=\frac{I\left(I+1\right)}{2}-N_{r}. \end{array}$$

The equation shows that the size of the phrase table is decided by the target length of the given sentence if the word alignment is the full word alignment, that is, the best and ideal quality word alignment. It is proven that the bidirectional training approach for word alignment can improve the quality of word alignment [8]. If the alignment is deduced from another direction (source side), similarly, the extracted phrases from source side can be represented by

(14) $$\begin{array}{c} N_{\text{min-}s}=\frac{J\left(J+1\right)}{2}-N_{r}. \end{array}$$

Equations (13) and (14) show that the number of phrases heavily affected by the length of source and target sentences. Given the best word alignment, if the length in the source sentence equals the target side (*I* = *J*), the phrases will be the minimal numbers (*N*_min-*s*_  or  *N*_min-*t*_). If not equal, the extracted phrases will be larger than this minimal value (*N*_min-*s*_  and  *N*_min-*t*_) after* intersection* and* union* operations during alignment process.

#### 3.1.2. Phrases Extraction from Worst Alignment

**Figure 5:**
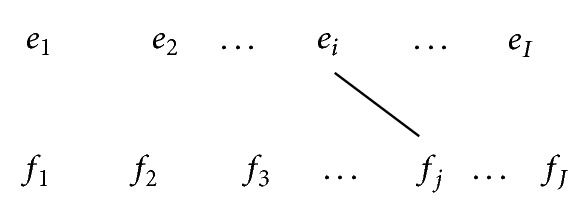
Sentences with single alignment link.

Suppose, only one word *f*_*j*_ in the source sentence is aligned to a target word *e*_*i*_ as shown in Figure 5. Note that unaligned words near the word *f*_*j*_ may lead to multiple possibilities, each proceeding and following words next to it can form a part of the phrase, and so does the neighboring words of *e*_*i*_ in target sentence. In other words, if we know the different extracted situations in each source and target sentence, and then total counts of phrase pairs can be derived by the product of the two numbers.  Algorithm 1 describes the different phrases extraction scenarios according to different positions in the source sentence.

Obviously, there are total *i* words that can start with (*e*_1_, *e*_2_, *e*_3_,…, *e*_*i*_). Now, in target sentence consisting of odd/even words, the phrase pairs *f*(*i*) in different position are

(15) $$\begin{array}{c} f\left(i\right)=i\left(I-i+1\right)=i\left(I-i\right)+i. \end{array}$$

The phrases *g*(*j*) in the source side can be calculated in a similar way:

(16) $$\begin{array}{c} g\left(j\right)=j\left(J-j+1\right)=j\left(J-j\right)+j. \end{array}$$

Based on (15) and (16), the total number of phrase pairs *N*_max⁡_ is given by:

(17) $$\begin{array}{c} N_{\max}=\left[i\left(I-i\right)+i\right]\left[j\left(J-j\right)+j\right], \end{array}$$

where, 0 ≤ *i* ≤ *I* − 1 and 0 ≤ *j* ≤ *J* − 1.

Obviously, (15) and (16) accord with* symmetric distribution*, and the maximal value will be achieved in the middle position. Given a sentence consisting of *L* words, a formal description to the middle position in the sentence can be presented by

(18) $$\begin{array}{c} {\text{word}}_{\text{mid}}\{\begin{array}{c} \frac{1+L_{\text{odd}}}{2}\\ \frac{L_{\text{even}}}{2}\;\text{or}\;\left(\frac{L_{\text{even}}}{2}+1\right) \end{array}. \end{array}$$

We can see that it gives (17) the maximal value in the middle position in a given sentence; that is, the nearer to the middle word, the more phrase pairs can be produced. Take the Figure 2 as an example, there are 18 phrases based on the full word alignments, while the phrases will be 240 when only the fifth word (*do*) in the source sentence aligns to the third word (**做**zuo) in the target side. From the view of size of phrase table, the calculation value from (17) is much larger than that from (13). That proves that the better quality of the word alignment, the fewer phrases will be.

Note that there will be some share phrases extracted from the* full word alignment* and only* single word alignment*, which leads to the phenomenon that there are still some translation abilities even with only one alignment point under the existing phrase extraction algorithm. For instance, phrase pairs (*e*_1_ *e*_2_ ⋯ *e*_*i*_ | *f*_1_ *f*_2_ *f*_3_ ⋯ *f*_*j*_),  (*e*_2_ ⋯ *e*_*i*_ | *f*_2_ *f*_3_ ⋯ *f*_*j*_),…, (*e*_*i*_ | *f*_*j*_) are the share ones, when given a phrase such as *f*_1_ *f*_2_ *f*_3_ ⋯ *f*_*j*_, both the phrase tables generated from both alignment types can possibly translate it right to *e*_1_ *e*_2_ ⋯ *e*_*i*_.

### 3.2. Phrase Table & Machine Translation

Nowadays, the key step of the process of phrase-based machine translation (SMT) involves inferring a large table of phrase pairs that are translations of each other from a large corpus of aligned sentences. The set of all phrase pairs, together with estimates of conditional probabilities and other useful features, is called Phrase Table (PT), which is applied during the decoding process [29].

Nevertheless, phrases in the PT are very dependent from the system that uses them. Some phrases might be present in the PT but never be used in translations because they are either ranked too low or erroneous translations, for example. In other words, there are too many redundant phrases in the PT. Such situation leads many researches to propose different techniques to reduce the size of the phrase table. This includes the methods as introduced in Section 2. These works can be treated as the research on the relationship between phrase table and machine translation.

All the works presented in Section 2 show that the translation results can be achieved from smaller phrase pairs, with several times increase in translation speed with little or no loss in translation accuracy. As reported by [32],* count-based* and* significance-based* pruning techniques can result in larger savings between 70% and 90%, while the* entropy-based *pruning approach can reduce consistently the entries between 85% to 95% of the phrase table. In [29], they even pointed out that the pruning rate can reach to 98% using a* bilingual segmentation* method with finite state transducers. More details about the pruning rates and related translation qualities (measured by BLEU [33]) are shown in Table 2, where the BLEU column is to record the maximal decrease (−) rate from the 100% size of phrase table to the maximal pruning rate of the phrase table.

The pruning rates presented above are counted from an empirical or experiment result. We can also estimate the pruning rate from a theoretical aspect. In practice, we also notice that the phrases extracted from the middle word alignment point include most of the phrases that are extracted from the full word alignment. All the phrases excluding the ones extracted from the full word alignment can be removed. In other words, a rough pruning rate can be calculated from the ration of phrases extracted from best alignment (*N*_min⁡_) and worst alignment (*N*_max⁡_):

(19) $$\begin{array}{c} R_{\text{pruned}}=1-\frac{N_{\text{min-}t}}{N_{\max}}. \end{array}$$

If we want to quickly estimate how many percentage of phrases can be pruned in a given corpus, the average statistics of the given corpus can be used as in (20). This equation shows that the pruning rate is not only decided by the sentence length in a parallel corpus but also affected by the alignment position. Consider

(20) $$\begin{array}{c} R_{\text{pruned}}=1-\frac{I_{\text{average}}\left(I_{\text{average}}+1\right)/2}{\left[i\left(I_{\text{average}}-i\right)+i\right]\left[j\left(J_{\text{average}}-j\right)+j\right]}. \end{array}$$

In reality, the numerator trends to bigger and the denominator will be smaller, so the pruning rate will be smaller than the calculation value. When the alignment position is in the middle position (middle alignment), there will get the maximal pruning rates as shown in (21). Based on the equation, we can estimate the pruning rate range based on different word alignment position in European corpus [46].  Table 3 shows the statistics of the WMT 2006 and Table 4 compares different pruning rates reported in researchers' paper [22, 32] and the calculation ranges based on (20). From which we can see that the calculation result is very close to the experiment results presented in [32]. The authors concluded their findings that “*the novel entropy-based pruning often achieves the same Bleu score with only half the number of phrases”*. A very bold assumption is that most phrases (at least half) can be removed from the original extracted phrases. Consider

(21) Rmax⁡=1−Iaverage(Iaverage+1)2×([imid(Iaverage−imid)+imid]  ×[jmid(Javerage−jmid)+jmid])−1.

We have seen that the phrase table pruning rate is strongly affected by the length and word positions in the source and target sentences. As reflected in the pruning equation (20), the phrase table might be more accurately pruned based on some features (e.g., phrase length, phrase cooccurrence) in the bilingual and monolingual corpus. The researchers in [27] reduced the phrase table based on the* significance testing* of phrase pair cooccurrence in* bilingual* corpus, while He et al. [38, 39] pruned the phrase pairs in terms of phrase frequency in the source side using* key phrases* extracted from a* monolingual* corpus. We want to make full use of the advantages of the two approaches: the* key phrases* can be used to check whether the extracted phrases are often used in practice and the* significant testing* can be used to measure the quality of the extracted phrase pairs. Our methods want to consider the features both for the source and target phrase.

The basic metrics for phrases that are often used in practice is the frequency that a phrase appears in a monolingual corpus. The more frequent a phrase appears in a corpus, the greater possibility the phrase may be used. However, longer phrase pairs will be removed in this way because of the fact that those phrases seem to appear rarely in a monolingual corpus.

The *C*-value is a measurement of automatic term recognition which can be used to solve this problem as described in [40]. The method not only fully considers the frequency information, such as frequencies of a phrase and a subphrase appearing in longer phrases, but also is an efficient algorithm.

In this algorithm (as shown in Algorithm 2), 4 factors (*L*, *F*, *S*, *N*) can be used to determine if a phrase *p* is a key phrase [38]:

- *L*(*p*), the length of *p*;
- *F*(*p*), the frequency that *p* appears in a corpus;
- *S*(*p*), the frequency that *p* appears as a substring in other longer phrases;
- *N*(*p*), the number of phrases that contain *p* as a substring.

Given a monolingual corpus, key phrases can be extracted efficiently according to Algorithm 2. Firstly (line 1), all possible phrases are extracted as candidates of key phrases (e.g., extracted phrase table from Moses [41]). Secondly (line 3 to 7), for each candidate phrase, *C*-value is computed according to the phrase appearing by itself (line 4) or as a substring of other long phrases (line 6). The *C*-value is in direct proportion to the phrase length (*L*) and occurrences (*F*, *S*), but in inverse proportion to the number of phrases that contain the phrase as a substring (*N*). This overcomes the limitations of frequency measurement. A phrase is regarded as a key phrase if its *C*-value is greater than a threshold *ε*. Finally (line 11 to 14), *S*(*q*) and *N*(*q*) are updated for each substring *q*.

The algorithm is originally used for pruning the rule table extracted from the hierarchical phrase-based SMT [42]. However, the idea can be also used for phrase-based SMT [1, 41] perfectly. After this filter step, a filtered phrase table called KP will be generated.

The phrases in the phrase table KP are all possibly used phrases in practice. However, there are still some noises in the KP table, for example, the source phrase occurring in source sentences and the target phrase occurring in the target sentences are not well matched; that is, the phrase translation is not good enough. To overcome this case, it is convenient to construct a two by two contingency table that tabulates the sentence pairs where the two types of matches occur and do not occur [21, 22, 43]. The calculation result is also called *P*-value as introduced in Section 2. A bilingual corpus constraint can be added to filter the noise phrases as presented in Algorithm 3. After this step, a large pruning rate phrase table (called *P*_*PT*) will be generated.

### 3.3. Word Alignment & Machine Translation

Based on the discussion in Sections 3.1 and 3.2, we can find that the size of phrase table is heavily affected by the quality of word alignment: the better the word alignment, the smaller size of the phrase table. Besides this, the machine translation performance is not directly decided by the size of the phrase table; at least 50% phrases can be removed without hurting the performance of the machine translation. Experiments show that the quality of phrase translation plays more important role to the final translation quality in practice. Therefore, it is necessary to improve the quality of word alignment under existing phrase extraction algorithm from word alignment.

Now, it is a little easier to talk about the relationship between word alignment and machine translation. The translation of sentence is directly derived from the phrase table, while the phrase table is extracted from word alignment. In other words, the quality of word alignment will affect the performance of machine translation through phrase table. That is to say, the word alignment quality will affect the translation performance a lot. However, the works listed in [8, 18, 19] show that there is not always significant affection to the translation result with improved word alignment quality.

We do not agree with the conclusion. Firstly, either the training or testing corpus they used in their experiments is limited in a small size or the improvement of the word alignment quality is not significant (AER decrease between 0.1 and 0.2). Secondly, we think it is the existing phrase extraction algorithm that leads to the not obvious result. We have pointed out in the last part of Section 3.1 that even the worst alignments are used, the extracted phrase table still have some ability to translate some input phrases. This is because of* the share phrases* extracted from the only one alignment link. The only one word alignment can translate some input phrases or sentences, not mentioning the more word alignment links.

We will discuss the situation with different word alignment in next section, where the machine translation affected by the best word alignment and worst word alignment will be fully surveyed. After that we can see that there are at least three advantages to improve the quality of word alignment as follows.

- Better alignment will extract fewer phrase pairs and keep a manageable size of phrase table.
- Better alignment will reduce the decoding time when searching the most possible translation from the phrase table.
- Better alignment will produce better quality of word or phrase level translation.

## 4. Experiments and Discussions

In order to evaluate the conclusions drawn in the previous section, some experiments are carried out using Moses toolkit [41], which provides a complete package of the required modules for training and generating the translation of texts.

### 4.1. Experiment Settings

**Figure 6:**
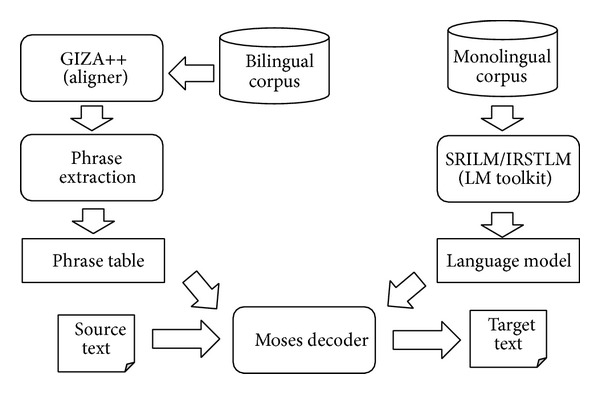
Moses' main modules and models.

Like other SMT approaches, Moses toolkit requires two models (as shown in Figure 6): the Language Model (LM) and the Translation Model (TM). In Moses, LM is usually created by the external toolkits SRILM [44] or IRSTLM [45]. In our experiment, the IRSTLM toolkit is used for the large training corpus. Before training, all the tokenization for languages in European corpus [46] use the integrated scripts in Moses, and the segmentation for Chinese adapts the Stanford Chinese segmenter [47] using CTB (Chinese Tree Bank) standard [48, 49]. The entire language model is trained by* five*-gram models.

The phrases are extracted from the results generated from GIZA++ [8]. The word alignment was trained with* ten* iterations of IBM model 1 and model 4 [34] and* six* iterations of the HMM alignment model [50].

To extract all the possible phrases to verify (13) and (17), the maximal phrases are limited to 30. This can extract most phrases in terms of word alignments and make us focus on the relations among word alignment, phrase table, and machine translation.

In order to better reveal relationships between alignment and phrase table to the translation quality,* leftmost*,* middle,* and* rightmost* word alignment are extracted from the original full alignment in the worst alignment situation (i.e., only one alignment link). Here the* leftmost*,* middle,* and* rightmost* word alignment indicate the word alignment link that aligned to the* first*,* middle,* and* end* word in the source language side.

### 4.2. Corpus Information

Three language pairs in WMT 2006 shared translation task are chosen as the training data: French-English (fr-en), German-English (de-en), and Spanish-English (es-en). These three language pairs are used to carry out the experiments for proving the equation deduced in Section 3 and relationship between word alignment and machine translation.  Table 3 shows the statistics of original corpus [46]. However, the number of phrases extracted from the only one link is too many to be allowed in our server, so the first 100,000 European corpus sentences (statistics shown in Table 5) are chosen to prove the availability of the equations between word alignment and phrase table.  Table 6 presents the statistics of 3,064 testing data from WMT 2006 for the same English sentences.

To test our algorithm for pruning the phrase table, a large Chinese-English parallel corpus is brought in. The large corpus consists of two parts: 3,289,497 sentences are from the CWMT 2013 [51] and 4,157,556 sentences are from UM-Corpus [52, 53]. After removing repeat and mismatched sentences in the combined two parts, there are left 7,445,190 sentences and the statistics of the combined parallel corpus are presented in Table 7. Note that the statistics of Chinese sentences are counted both in character level (each Chinese character is treated as one token, Character_CE_  in Table 7) and word level (segmented by Stanford CTB segmenter, presented by CTB_CE_).

The UM-Corpus also contains English, Chinese, and Portuguese monolingual corpora. In this experiment, the English and Chinese corpora are used. There are 35,938,697 English sentences from news domain and more than 70 million Chinese sentences from China portal webpage. The statistics about the two corpora are shown in Table 8.

The 5000 sentences of test data (named UM-Testing) are designed according to domains in the UM-Corpus. There are 1,500 sentences for* news*, 500 sentences for* laws*, 500 sentences for* novels*, 600 sentences for* thesis*, 700 sentences for* education*, 600 sentences for* science,* and 600 for* subtitle*. The statistics of the testing data are shown in Table 9.

### 4.3. Equation of Word Alignments versus Phrases

The calculation for number of phrases requires the full word alignment links. Full word alignment, in other words, is the best quality of word alignment. It is, obviously, very time-consuming if we manually edit the large word aligned sentences. It is already observed in the past that generative models used for statistical word alignment create alignments of increasing quality as they are exposed to more data [19]. The intuition behind this is simple; as more cooccurrences of source and targets words are observed, the word alignments are better. Following this intuition, if we wish to increase the quality of a word alignment, we allow the alignment process access to extra data which is used only during the alignment process and then removed. If we wish to decrease the quality of a word alignment, we divide the parallel corpus into pieces and align the pieces independently of one another and finally concatenate the results together. In our experiments, we use the entire WMT 2006 corpus as training data firstly, and then the first 100,000 alignments are chosen as the full word alignments.

Also, the* leftmost*,* middle,* and* rightmost* word alignment point based on the* full word* alignment are obtained based on the results generated by GIZA++.

The minimal and maximal sizes of the phrase table extracted from the* full* and* middle* word alignment and the differential rates are shown in Table 10. The differential rate is calculated by

(22) $$\begin{array}{c} R_{\text{diff-min}\;}=\frac{\left|N_{\min}-N_{\text{moses-min}}\right|}{N_{\min}}, \end{array}$$

(23) $$\begin{array}{c} R_{\text{diff-max}}=\frac{\left|N_{\max}-N_{\text{moses-max}}\right|}{N_{\max}}, \end{array}$$

where the *N*_moses-min_ and *N*_moses-max_ indicate the minimal and maximal phrases extracted from Moses.

From Table 10 we can see that the actual number of phrases is close to the calculation result. With the small differential rate, the (13) and (17) can be available to the practical experiments. The difference may come from two reasons: (1) the assumption of full word alignment produced from the GIZA++ may contain too many noises; (2) equations (13) and (17) do not consider repetitions of the extracted phrases.

### 4.4. Phrase Table versus Machine Translation

We have shown that the there is no direct relationship between the size of phrase table and machine translation performance. As reflected in (17) and Table 4, at least half of the phrases can be removed without hurting too much the performance of machine translation. In this part, we want to use the large Chinese-English corpus to test our pruning algorithm based on monolingual and bilingual corpus.

According to our experience, different thresholds will generate different size of phrase table and result in different performance of translation. Therefore, one of the key steps is to choose the pruning threshold in Algorithms 2 and 3. Firstly, a baseline threshold is calculated from the logarithm of sentences of the given corpus (*N*), that is, log⁡(*N*). For the *C*-value threshold, the baseline threshold is about 25 (log⁡(3.6∗107)). For the *P*-value threshold, the negative logarithm will be chosen. Because the probabilities involved are incredibly tiny, we will work instead with the negative of the natural logs of the probabilities. For example, instead of selecting phrase pairs with a *P*-value less than exp⁡(−15), we will select phrase pairs with a negative-log-*P*-value greater than 15. The baseline threshold for *τ*_*F*_ is 45 (log⁡(7.4∗106)). Secondly, the different thresholds will increase or decrease than the baseline threshold. The affection to the phrase table and BLEU scores based on different thresholds is shown in Table 11.

From Table 11, we can see that nearly 98% phrases can be discarded with monolingual and bilingual corpora, while the translation quality does not become worse too much (only decrease 2.35). Experiments show that about 85% of the phrase table is reduced and can generate the best translation quality, which not only again proves the similar results as in other researchers [3, 21, 32, 38], but also indicates that most phrases are not needed at all. The best translation can be achieved on the size of about 15% to 30% of the original phrase table. That is to say, only a small fraction of phrases are the essential elements, while major of the extracted phrases are spurious, and in some sense, they are redundancy and should be discarded.

### 4.5. Word Alignment versus Translation Quality

In order to investigate the affection to the translation performance based on different word alignment types and phrase table size, we selected five different types of word alignments to generate the phrase table and then measure the translation quality by BLEU metric: the full word alignment points (*A*_full_), the leftmost word alignment (*A*_*l*most_), the sure alignment point (*A*_sure_), the middle word alignment point (*A*_mid_), and the rightmost word alignment point (*A*_*r*most_).

**Figure 7:**
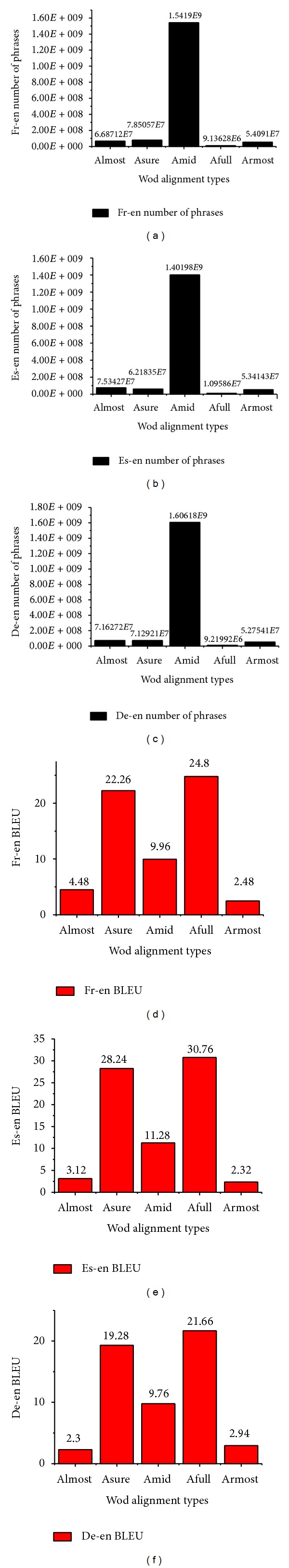
Number of phrases extracted from different word alignment points and their BLEU.

Clearly, the *A*_full_ is the best quality of word alignment and followed by *A*_sure_, which is the only 1-to-1 alignment. The *A*_*l*most_, *A*_mid_, and *A*_*r*most_ are much worse ones, which is only one alignment link. As mentioned previously, all these alignment points are obtained after getting the results generated by GIZA++. The experiment is carried out in WMT 2006 corpus. Based on the different alignment types, the number of phrases extracted and the corresponding translation BLEU are presented in Figure 7 and Table 12 separately.

The histograms show that there is not any direct proportion relationship between the translation performance and the number of extracted phrases. The numbers in Figure 7 show that German-English language pairs can generate the most phrases, while the translation quality is not the best. Although the fewest phrases produced from the Spanish-English language pairs, the best translation performance can be obtained. Explained by (17), more phrases usually lead to worse word alignment quality or it can be said that there are too many unaligned words in the produced alignment results. The results in Figure 7 and Table 12 show that (1) the full word alignment will contribute most to the translation performance and there will not be too much decrease when only the sure alignment is provided; (2) however, the* first* and* last* word alignment will not produce too high translation quality; (3) although the middle position word alignment can extract the most phrases, the quality of the translation is not the best and it seems that middle word alignment can contribute more to the finally translation.

In fact, the translation from the only one link word alignment should be higher. After all, the default full word alignments have too many noises, which trained from a large training corpus without manual edit.

## 5. Conclusions

In this paper, some investigations are made about the relationship among word alignment, phrase table, and machine translation. After deducing a formula for estimating the size of the phrase table, a maximal phrase pruning ratio can be calculated based on the average length of the given corpus and word alignment positions. Finally, translation performance based on different word alignment types is compared.

Equations (13) and (17) provide a simple way to predict the size of the phrase table in advance based on the generated word alignment points. This is beneficial for evaluating how much hardware resources are required to train the model when the size of the parallel corpus is huge.

Experiment results show the affection to the translation performance is significant if the AER decreases or increases too much. There is no direct proportion relationship between the translation performance and the number of extracted phrases. In other words, most of the phrases are not needed at all, only phrases extracted from the full word alignment are enough. It can be seen from our corpus-motivated pruning method that only 15%–30% phrases are needed for the basic translations and most phrases in the phrase table are noises to the final translation.

Based on the existing phrase extraction algorithm from word alignment, better word alignment will be very helpful to the final translation. There are at least three advantages with a better quality of word alignment even without the existing phrase extraction algorithm improved as follows.

- Better alignment will extract fewer phrase pairs and keep a manageable size of phrase table.
- Better alignment will reduce the decoding time when searching the most possible translation from the phrase table.
- Better alignment will produce better quality of word or phrase level translation.

The next work we want to focus on is to add the corpus-motivated pruning technique into the translation models; that is, the phrases will be filtered during the training time, not after generating the phrase table.

## Figures and Tables

**Algorithm 1:**
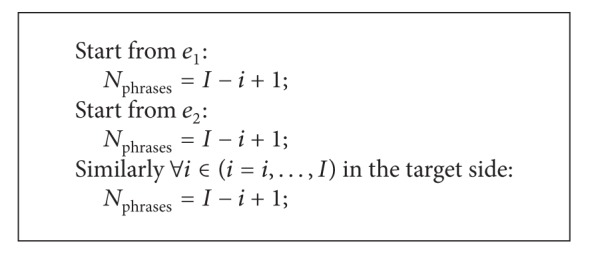
Phrase extraction based on the only one alignment point: *e*_*i*_↔*f*_*j*_.

**Algorithm 2:**
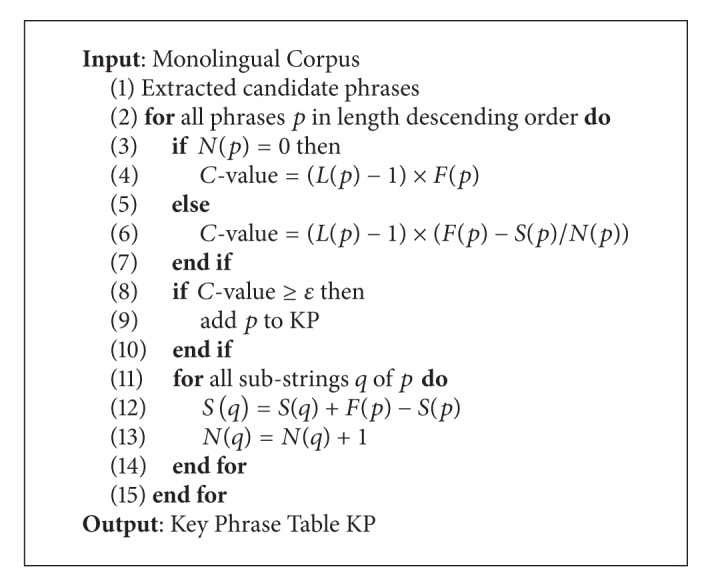
Key phrase extraction from monolingual corpus.

**Algorithm 3:**
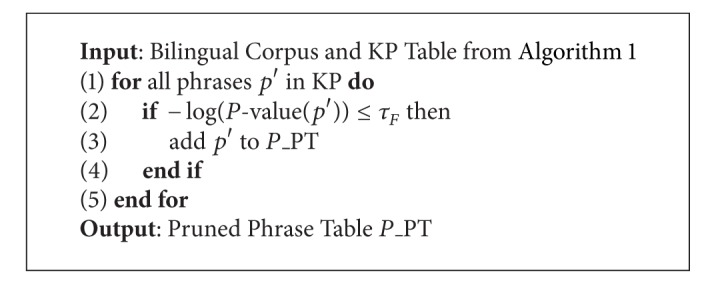
Algorithm for key phrase pruning.

**Table 1:** Contingency table.

	$\overset{-}{e}$	${\overset{-}{e}}^{\prime }$	
$\overset{-}{f}$	$N\left(\overset{-}{f},\overset{-}{e}\right)$	$N\left(\overset{-}{f}\right)-N\left(\overset{-}{f},\overset{-}{e}\right)$	$N\left(\overset{-}{f}\right)$
${\overset{-}{f}}^{\prime }$	$N(\overset{-}{e})-N\left(\overset{-}{f},\overset{-}{e}\right)$	$N-N\left(\overset{-}{f}\right)-N(\overset{-}{e})-N\left(\overset{-}{f},\overset{-}{e}\right)$	$N-N\left(\overset{-}{f}\right)$
	$N(\overset{-}{e})$	$N-N(\overset{-}{e})$	*N*

**Table 2:** Pruned phrase table and translation quality.

Pruning methods	Languages	Pruning rates	BLEU (Δ)
Count/significant	fr-en	64.6%~85.9%	−0.1
	es-en	67.9%~88.1%	
	de-en	62.7%~84.1%	

Entropy	fr-en	85%~95%	−1.0
	es-en	85%~95%	
	de-en	85%~95%	

Bilingual-segment	fr-en	—	−1.2
	es-en	98%	
	de-en	94%	

**Table 3:** Training data statistics: WMT 2006.

Language pairs	Sentences	Ave. source len.	Ave. target len.
fr-en	688,031	22.67	20.07
es-en	730,740	21.52	20.83
de-en	751,088	20.31	21.37

**Table 4:** Comparison between theoretical estimation results and other pruning techniques results.

Language pairs	*j*_mid_	*i*_mid_	*R*_pruned_
fr-en	12.34	11.04	53.5%~98.6%
es-en	11.26	11.42	49.3%~98.4%
de-en	11.16	11.19	44.9%~98.3%

**Table 5:** Statistics of the first 10,000 WMT 2006 corpus.

Languages	Ave. source len.	Ave. en len.
fr-en	22.89	20.15
es-en	21.71	20.94
de-en	20.55	21.42

**Table 6:** Statistics of test data (3,064 sentences).

Languages	Ave. source len.	Ave. en len.
fr-en	32.95	27.81
es-en	29.94	27.81
de-en	26.92	27.81

**Table 7:** Statistics of CMWT 2013 + UM-Corpus.

Languages	Tokens	Average length	Vocabularies
English	152,161,233	19.37	1,655,080
Character_CE_	229,110,265	29.16	397,442
CTB_CE_	123,917,395	15.78	1,331,505

**Table 8:** Statistics of English & Chinese corpora.

Languages	Tokens	Average length	Vocabularies
English	804,584,844	24.64	2,357,374
Chinese	3,007,196,322	33.43	1,533,343

**Table 9:** Statistics for EC-Corpus testing data.

Languages	Tokens	Average length
English	118,802	23.76
Character_CE_	153,705	30.74
CTB_CE_	118,499	23.70

**Table 10:** Phrases comparison between Moses extraction and calculation result.

Phrase length	*K* = 30					
Languages	*N*_moses-min_	*N*_min⁡_	*R*_diff-min_	*N*_moses-max_	*N*_max⁡_	*R*_diff-max_
fr-en	9,136,283	9,080,307	0.006	1,541,902,790	1,544,021,509	0.001
es-en	10,958,551	10,895,266	0.006	1,401,977,185	1,489,422,170	0.058
de-en	9,219,921	8,929,095	0.033	1,606,176,665	1,686,043,482	0.047

**Table 11:** *C*-value and *P*-value threshold effect on the phrase table size and BLEU scores.

Thresholds		UM-Corpus + CWMT 2013	
*C*-value *ε*	−log⁡(*P*-value) *τ*_*F*_	Phrase table (%)	BLEU (%)
0	0	100	28.13
10	20	83.6	28.09
25	45	52.5	27.98
100	60	29.7	28.11
200	100	14.3	**28.27**
400	200	2.02	25.78

**Table 12:** Translation performance on different word alignment types.

Languages	BLEU (3,064 sentences)				
	*A*_full_	*A*_*l*most_	*A*_sure_	*A*_mid_	*A*_*r*most_
fr-en	24.80	4.48	22.26	9.96	2.48
es-en	30.76	3.12	28.24	11.28	2.32
de-en	21.66	2.30	19.28	9.76	2.94
